# Vaginal microbiome differences between patients with adenomyosis with different menstrual cycles and healthy controls

**DOI:** 10.1186/s12866-024-03339-9

**Published:** 2024-07-27

**Authors:** Zangyu Pan, Jun Dai, Ping Zhang, Qianhui Ren, Xinyu Wang, Shumin Yan, Hao Sun, Xue Jiao, Ming Yuan, Guoyun Wang

**Affiliations:** 1 Department of Obstetrics and Gynecology, Shandong Provincial Hospital, Jinan, 250000 Shandong China; 2 Medical Integration and Practice Center, Cheeloo College of Medicine, Shandong University, Jinan, 250000 Shandong China; 3 JiNan Key Laboratory of Diagnosis and Treatment of Major Gynaecological Disease, Jinan, 250000 Shandong Province China; 4 Gynecology Laboratory, Shandong Provincial Hospital, Jinan, 250000 Shandong Province China; 5 Gynecology Laboratory, Medical Science and Technology Innovation Center, Shandong First Medical University & Shandong Academy of Medical Sciences, Jinan, 250000 Shandong Province China; 6 Qilu Hospital of Shandong University, Jinan, 250000 Shandong Province China

**Keywords:** Adenomyosis, Vaginal microbiome, Menstrual cycles

## Abstract

**Background:**

Adenomyosis is a commonly observed benign gynecological disease that affects the quality of life and social psychology of women of childbearing age. However, because of the unknown etiology and incidence of adenomyosis, its pathophysiological mechanism remains unclear; further, because no noninvasive, accurate, and individualized diagnostic methods are available, treatment and efficacy evaluations are limited. Notably, the interaction between the changes in the microecological environment of the female reproductive tract and human immunity, endocrine, and other links leads to the occurrence and development of diseases. In addition, the vaginal microbiome differs in different menstrual cycles; therefore, assessing the differences between the microbiomes of patients with adenomyosis and healthy individuals in different menstrual cycles will improve the understanding of the disease and provide references for the search for noninvasive diagnosis and individualized precision treatment of adenomyosis. This study aimed to explored the data of individuals in different menstrual cycles.

**Results:**

Differences in the vaginal microbiome between patients with adenomyosis and healthy individuals were observed. At phylum level, the relative abundance of *Firmicutes* in the adenomyosis group was higher than that in the control group, which contributed the most to the species difference between the two groups. At the genus level, *Lactobacillus* was the most dominant in both groups, Alpha-diversity analysis showed significant differences in the adenomyosis and control group during luteal phase (Shannon index, *p* = 0.0087; Simpson index, *p* = 0.0056). Beta-diversity index was significantly different between the two groups (*p* = 0.018). However, based on Weighted Unifrac analysis, significant differences were only observed throughout the luteal phase (*p* = *0.0146*). Within the adenomyosis group, differences between women with different menstrual cycles were also observed. Finally, 50 possible biomarkers including were screened and predicted based on the random forest analyse.

**Conclusions:**

The vaginal microbiome of patients with adenomyosis and healthy individuals differed during menstrual periods, especially during the luteal phase. These findings facilitate the search for specific biological markers within a limited range and provide a more accurate, objective, and individualized diagnostic and therapeutic evaluation method for patients with adenomyosis, compared to what is currently available.

**Supplementary Information:**

The online version contains supplementary material available at 10.1186/s12866-024-03339-9.

## Introduction

Adenomyosis is a benign uterine myometrial lesion commonly found in women of reproductive age and is characterized by compensatory hypertrophy in the peripheral myometrium, with endometrioid glands and stroma found in the myometrium [1]. Pathological diagnosis after surgery is the gold standard for clinical diagnosis; however, the exact incidence and pathogenesis of adenomyosis remain unknown [2]. Studies have shown that a history of uterine surgery is a high risk factor for adenomyosis. For example, the incidence of adenomyosis in patients with the aforementioned surgical history is 1.5 times higher than in patients with a different history [3, 4]. In the treatment of adenomyopathy, in addition to surgical treatment, conservative programs are used to regulate endocrine and immune system functions. Diagnostic methods include magnetic resonance imaging (MRI), transvaginal ultrasonography, and CA125 test, however, no specific, individualized diagnostic method is available. Adenomyosis and other benign gynaecological diseases, such as uterine fibroids, endometriosis, and endometrial polyps, have a high comorbidity rate, and attributing specific symptoms to adenomyosis in clinical diagnosis and treatment is difficult.

The vagina is an important organ of the female lower genital tract and is an important habitat for microorganisms in the human body. *Lactobacillus* is the predominant bacterial species and is affected by various exogenous and endogenous factors; furthermore, the species composition of the vaginal microbiome has a strong dynamic change [5]. The vaginal microbiome is an important defence mechanism that regulates and maintains reproductive function and relative homeostasis in healthy environments. The stability of the microbiome can prevent the proliferation of symbiotic microorganisms and the colonization of pathogens [6]. Microorganisms affect the balance of the microenvironment through nutritional competition, intraspecific and interspecific signal transduction, metabolic pathways, and product interactions. The mechanism of microenvironmental imbalance remains unclear; however, this imbalance can disrupt normal homeostasis, resulting in certain pathological signs. The female upper reproductive tract was once considered a sterile environment; however, this theory has been challenged. The presence of microbiota in the endometrial microbiota [7] was confirmed by the isolation of microbiota from female endometrial aspirated fluid samples. Studies have shown that bacterial DNA can be detected in 95% of post-hysterectomy samples [8]. Microbial switching occurs in the female reproductive tract, and the microbiota of the upper and lower reproductive tracts work synergistically to regulate the uterine environment. With increasing age, synchronous changes in the microbiome of the uterus and vagina increasingly converge, showing a mutually parallel relationship. Animal studies have verified the damaging and protective effects of vaginal bacteria on the endometrium using microbiota transplantation techniques [9]. This also indicates that lower reproductive tract bacteria affect or directly interfere with the regulation of some benign and malignant diseases, to some extent, through certain mechanisms.

Initial research on vaginal microbes mainly relied on microscopy and microbial culture techniques; however, the vast majority of microorganisms in the physiological or natural environment are difficult to obtain through culture. Using bioinformatics, high-throughput sequencing and analysis technology were performed to minimise the dependence on bacterial culture technology used in the literature and enhance our understanding of the structure and function of the microbial community, as well as of the association between the bacterial community of this "non-visual organ" and benign and malignant diseases of the female reproductive system.

The 16S-rRNA is a subunit of ribosomal RNA. With improvements in sequencing technology, 16S-rDNA amplicon sequencing has become an important method to evaluate the microenvironment, structure, and composition [10–13]. As research progresses, sequencing platforms are updated and iterated. Relying on the upgraded Illumina NovaSeq sequencing platform, we compensated for the inefficiency of single-ended reading and realized double-ended sequencing; that is, small fragment libraries were built according to the characteristics of the amplified regions.

According to our review of the literature, no study has investigated the differences in the vaginal microbiome between adenomyosis patients with different menstrual cycles and healthy individuals. Therefore, this study aimed to elucidate the differences in the vaginal microbiota between women with and without adenomyosis, with different menstrual cycles. Our results provide a reference for the subsequent screening of characteristic biological markers, disease diagnosis, non-invasive precision treatment, and efficacy prediction based on microbial detection.

## Materials and methods

The case group in this study comprised patients with adenomyosis in the gynecological outpatient department of Affiliated Hospital of Shandong University from November 2021 to October 2022 were selected as the case group. They were evaluated by professional gynecologists, and adenomyosis was confirmed by ultrasound or magnetic resonance imaging (MRI). The control group comprised healthy individuals. The inclusion criteria were as follows: (1) 18–49 years old; (2) no unhealthy lifestyle; (3) Regular menstrual cycle; (4) non-pregnant, non-puerperal, non-lactation, not during the menstrual phase of the estrogen cycle; (6) pre-menopause. The exclusion criteria were as follows: (1) no medical history could be provided; (2) cervical intraepithelial lesions, cervical malignancies, vulva lesions and other HPV-related diseases; (3) virus or bacterial infection; (4) history and treatment of endocrine system diseases; (5) autoimmune diseases; (6) acute/chronic inflammation of the urogenital tract; (7) sexually transmitted diseases and infectious diseases; (8) malignant tumors; (9) history of sexual life, vaginal bleeding, vaginal douching, vaginal medication, sitting bath, pelvic bath, transvaginal examination 48 h before sampling; (10) history of use of antibiotics, antifungals, and hormonal treatments within 30 days before sampling; (11) intrauterine device implantation; (12) recent history of pelvic and abdominal surgery and intrauterine operation.

### Sample collection

The individuals who fulfilled the inclusion criteria had a clinical sample collected on the day of the clinical visit before they received a transvaginal gynecologic examination or gynecologic ultrasound. The posterior vaginal fornix was fully sampled using disposable sterile swabs. During the procedure, contact between the swab head and the speculum, vaginal wall, and other non-sampling sites was avoided. The swab head was cut off with sterile scissors and placed in a sterile centrifuge tube containing Amies culture medium (JINAN BABIO BIOTECHNOLOGY CO,.LTD.), and stored at -80 ℃ in the laboratory.

### Extraction of genome DNA

The genomic DNA of the sample is extracted by cetyltrimethylammonium bromide (CTAB) method. DNA concentration and purity was monitored on 1% agarose gels. According to the concentration, DNA was diluted to 1 ng/µL using sterile water. Using the diluted genomic DNA as a template, the V3-V4 region of 16S-rDNA gene was amplified. The primer sequence was as follows: ①F:CCTAYGGGRBGCASCAG; ②R:GGACTACNNGGGTATCTAAT (Phusion® High-Fidelity PCR Master Mix with GC Buffer, New England Biolabs,lnc.). Polymerase Chain Reaction (PCR) was performed using specific primers with Barcode and high-efficiency high-fidelity enzyme according to the selection of sequencing region to ensure amplification efficiency and accuracy. All PCR reactions were carried out with 15µL of Phusion® High-Fidelity PCR Master Mix (New England Biolabs); 2 µM of forward and reverse primers, and about 10 ng template DNA. Thermal cycling consisted of initial denaturation at 98℃ for 1 min, followed by 30 cycles of denaturation at 98℃ for 10 s, annealing at 50℃ for 30 s, and elongation at 72℃ for 30 s. Finally 72℃ for 5 min.

### Library construction and sequencing

Sequencing libraries were generated using TruSeq® DNA PCR-Free Sample Preparation Kit (Illumina, USA) following manufacturer's recommendations and index codes were added. The library quality was assessed on the Qubit@2.0 Fluorometer (Thermo Scientific) and Agilent Bioanalyzer 2100 system. At last, the library was sequenced on an Illumina NovaSeq platform and 250 bp paired-end reads were generated.

### Paired-end reads assembly and quality control

Paired-end reads was assigned to samples based on their unique barcode and truncated by cutting off the barcode and primer sequence. Paired-end reads were merged using FLASH (V1.2.7, http://ccb.jhu.edu/software/FLASH/) [14], which was designed to merge paired-end reads when at least some of the reads overlap the read generated from the opposite end of the same DNA fragment, and the splicing sequences were called raw tags. Quality filtering on the raw tags were performed under specific filtering conditions to obtain the high-quality clean tags [15] according to the QIIME(V1.9.1, http://qiime.org/scripts/split_libraries_fastq.html) [16] quality controlled process. The tags were compared with the reference database (Silva database, https://www.arb-silva.de/) [17] to detect chimera sequences, and then the chimera sequences were removed [18]. Then the Effective Tags finally obtained.

## Results

The study enrolled 43 patients with adenomyosis and 40 healthy people. There were no significant differences in demographic background between the two groups of participants (Table 1).

**Table 1:** Demographic data of the subjects

	Adenomyosis (*N* = 40)	Control (*N* = 40)	*P*-value
Age, years (mean ± SD)	39.81 ± 5.62	38.38 ± 5.51	0.243
BMI, kg/m^2^, (mean ± SD)	23.73 ± 2.81	22.32 ± 3.88	0.060
Gestation	2.19 ± 1.20	1.77 ± 1.07	0.055
Delivery	1.07 ± 0.67	1.00 ± 0.56	0.687
Menstrual cycle, days	26.88 ± 3.67	27.97 ± 2.15	0.066
Menstrual period, days	5.81 ± 1.33	5.47 ± 1.32	0.153

**Fig. 1:**
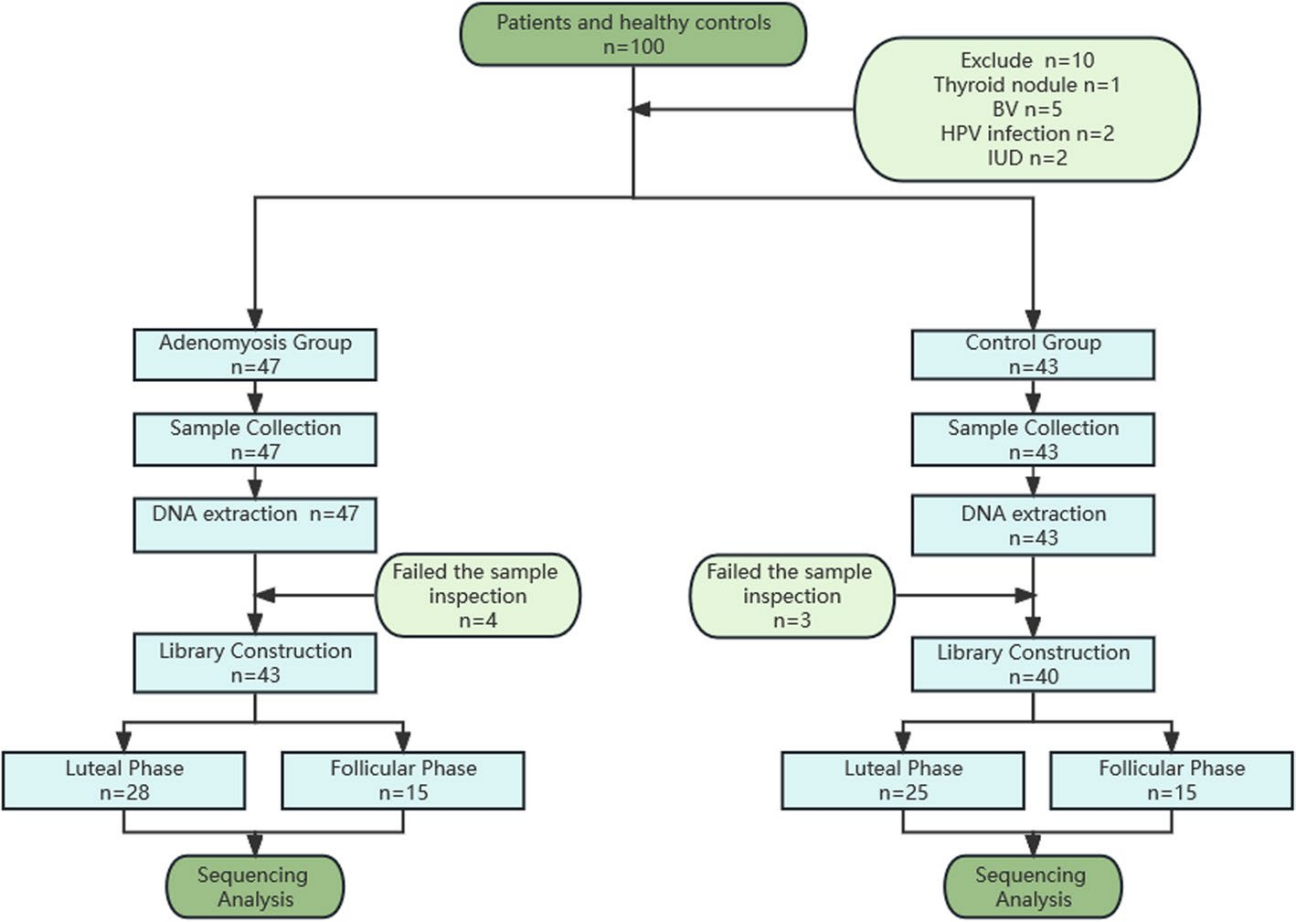
Study process

The vaginal samples were collected from all participants; however, 7 samples in total were excluded from the control group due to poor DNA quality after library quality check. Therefore, 83 samples were used in the subsequent analysis. (Fig. 1).

Next, the vaginal microbiota was analyzed using 16 s rDNA sequencing techniques. The Raw PE data sequenced by Illumina Novaseq were splicing and quality control to obtain Clean Tags, and then chimeric filtering was performed to obtain Effective Tags for subsequent analysis (S1 Table).

### Species relative abundances

**Fig. 2:**
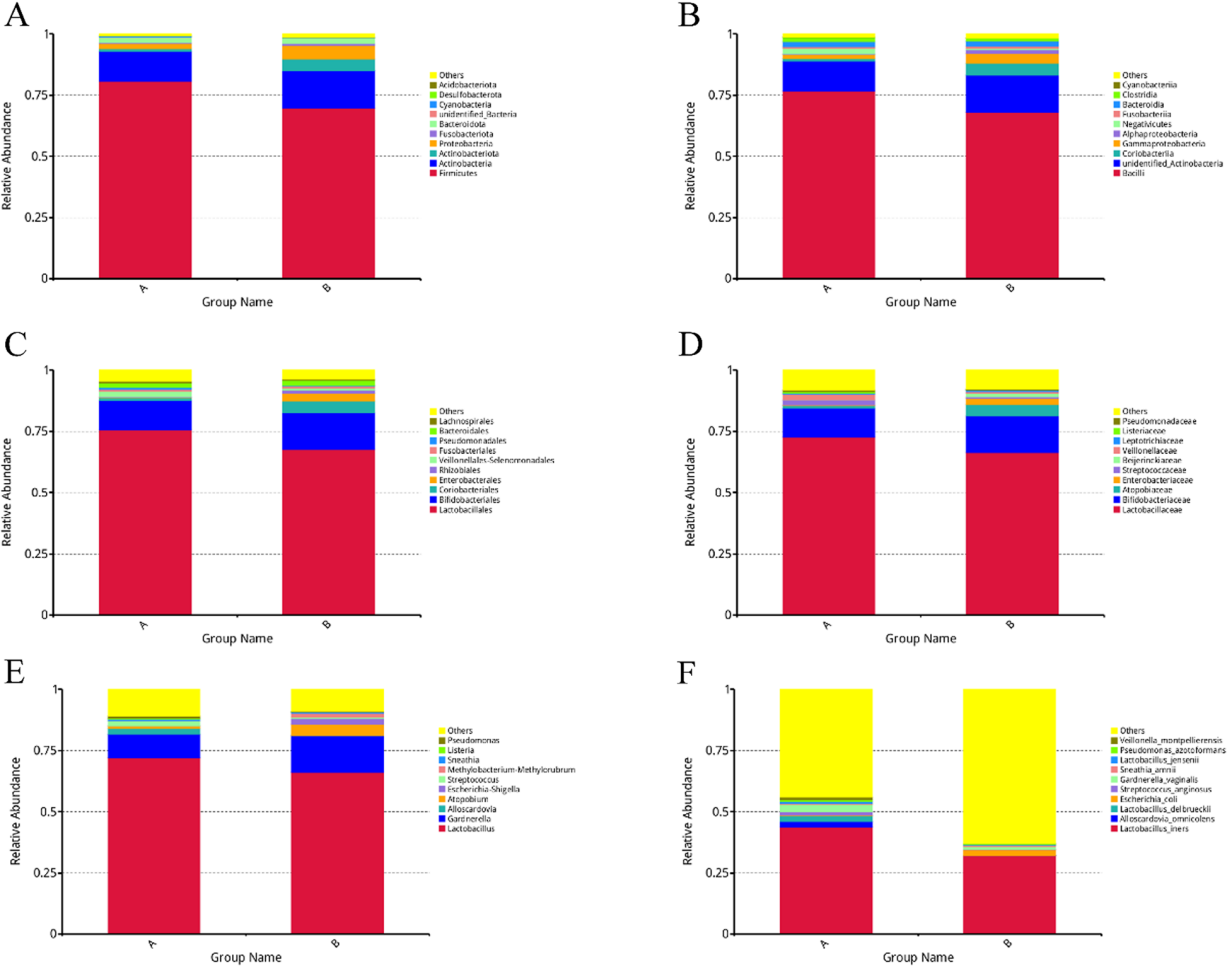
Taxonomy bar charts of vaginal microbiame at (**A**) phylum, (**B**) class, (**C**) order, (**D**) family, (**D**) genus and (**E**) species level

At phylum level, the relative abundance of *Firmicutes* in adenomyosis group was higher than that in control group (80.70% and 69.72% in adenomyosis and control groups). At the genus level, the *Lactobacillus* relative abundance in both adenomyosis group and control group was the highest (72.10% and 66.08%). But the relative abundance of *Gardnerella* and *Atopobium* in the adenomyosis group was lower than that in the control group (9.67% and 1.04% in adenomyosis and 14.95% and 4.69% in control groups); At the Species level, the *Lactobacillus_iners* abundance in the adenomyosis group was higher than that in the control group(43.74% and 32.14%), and showed a diversity of Lactobacillus, including *Lactobacillus_delbrueckii* and *Lactobacillus_jensenii* (Fig. 2).

### Different menstrual cycles

**Fig. 3:**
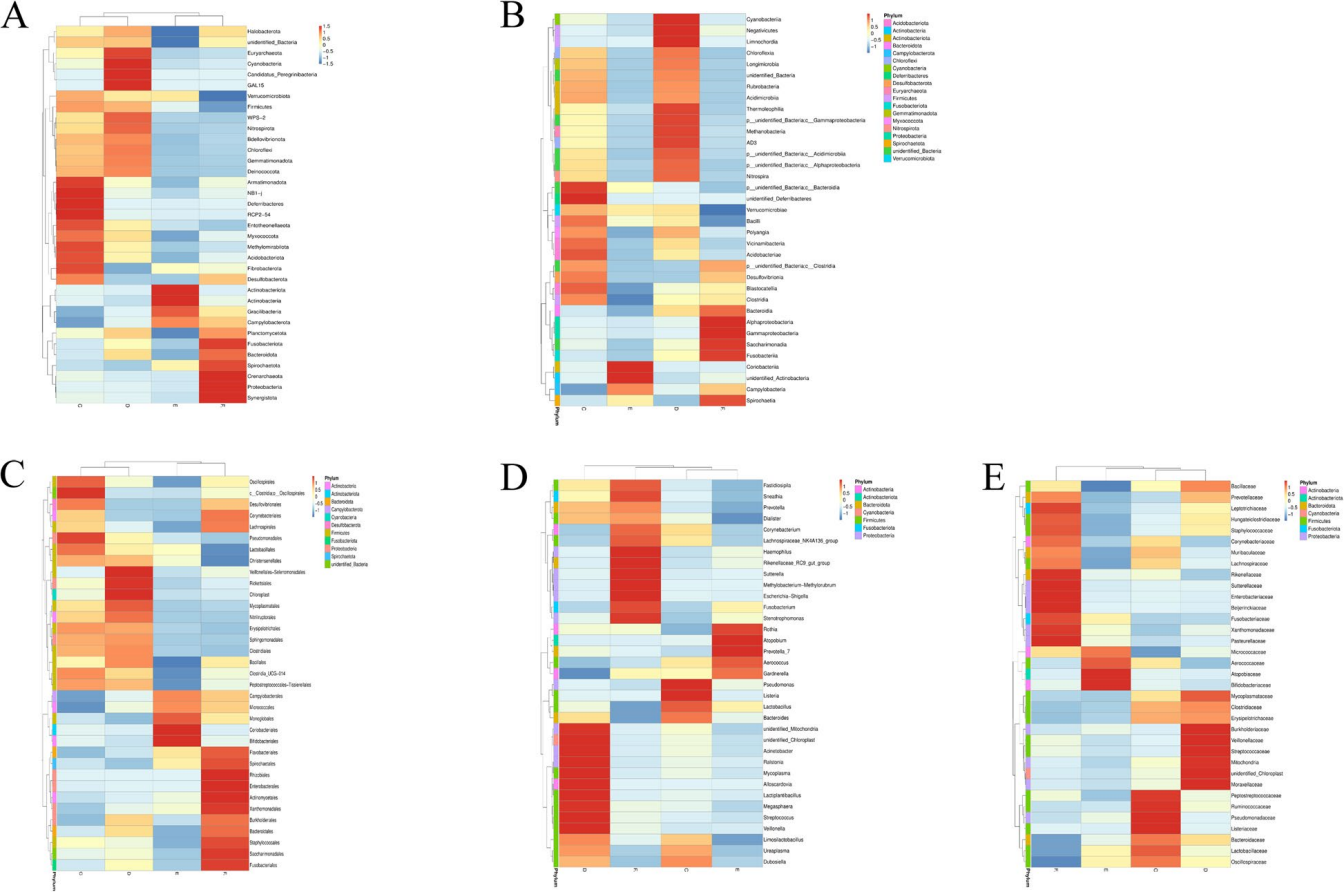
Heatmap of species abundance clustering during different menstrual cycles. The top 35 species with the average abundance of all samples of the same level and different groups are selected for clustering at (**A**) phylum, (**B**) class, (**C**) order, (**D**) family, (**D**) genus and (**E**) species level.The heatmap is drawn by heatmap package of R software, which is convenient to find the number or content of species in the sample

The top 35 species with the average abundance of all samples of the same level and different groups are selected for clustering, and the heatmap is drawn by heatmap package of R software, which is convenient to find the number or content of species in the sample (Fig. 3).

### Sample complexity analysis

In order to study the influence of menstrual cycle on vaginal microecology, we named all the samples in the luteal phase of the adenomyosis group as group C and the follicular phase as group D. All the samples in the luteal phase of the control group were named group E and group F. in the follicular phase.

**Fig. 4:**
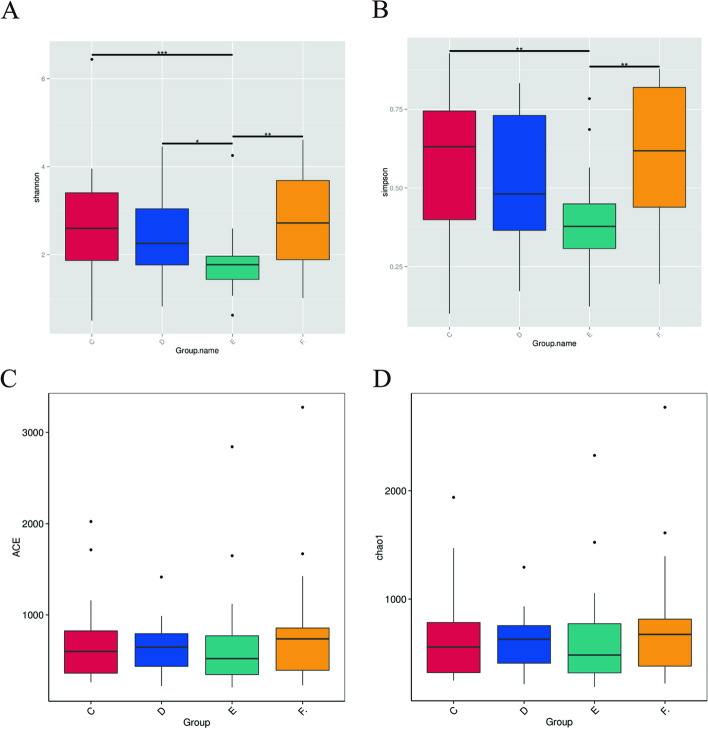
Alpha-diversity analysis. **A** shannon index, (**B**)Simpson index, (**C**) ACE index, (**D**) chao1 index. Alpha-diversity analysis indices for different samples at 97% consistency thresholds

**Fig. 5:**
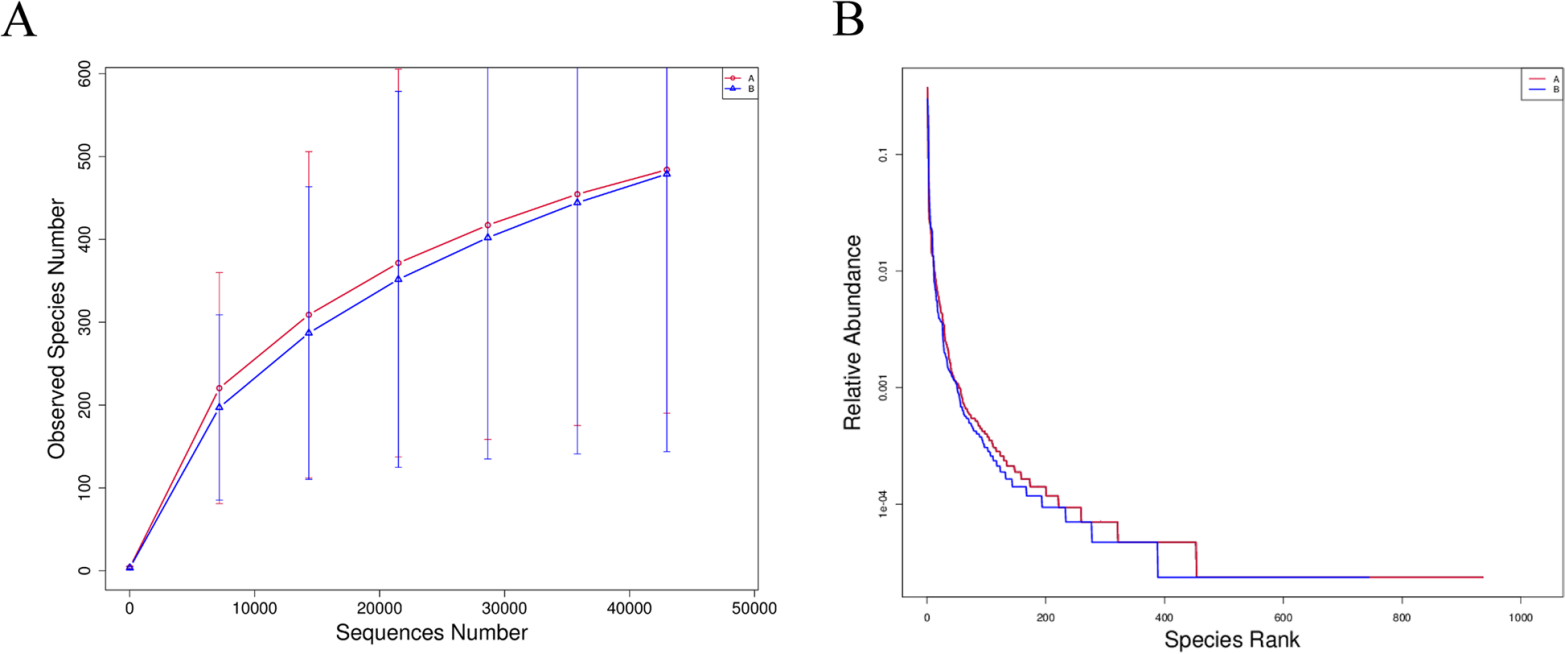
Rarefaction curve and Rank Abundance curve. In the (**A**) Rarefaction curve, horizontal coordinate is the number of sequencing strips randomly selected from a sample, and the vertical coordinate is the number of Operational Taxonomic Units (OTUs) that can be constructed based on the number of sequencing strips, which is used to reflect the sequencing coverage, and different samples are represented by different colored curves; in the (**B**) Rank Abundance curve, the horizontal coordinate is the serial number sorted by the abundance of OTUs, and the vertical coordinate is the relative abundance of the corresponding OTUs, and different samples are represented by different colored fold lines

Alpha-diversity analysis showed significant differences in the adenomyosis and control group during luteal phase (Shannon index, *p* = 0.0087; Simpson index, *p* = 0.0056), but we didn’t find the statistically difference in ACE and chao 1 index (Fig. 4). It was verified that the amount of sequencing data was progressive and reasonable, and more data would only produce a few new species, thus suggesting a uniform distribution of species (Fig. 5).

### Comparative analysis of multiple copies

**Fig. 6:**
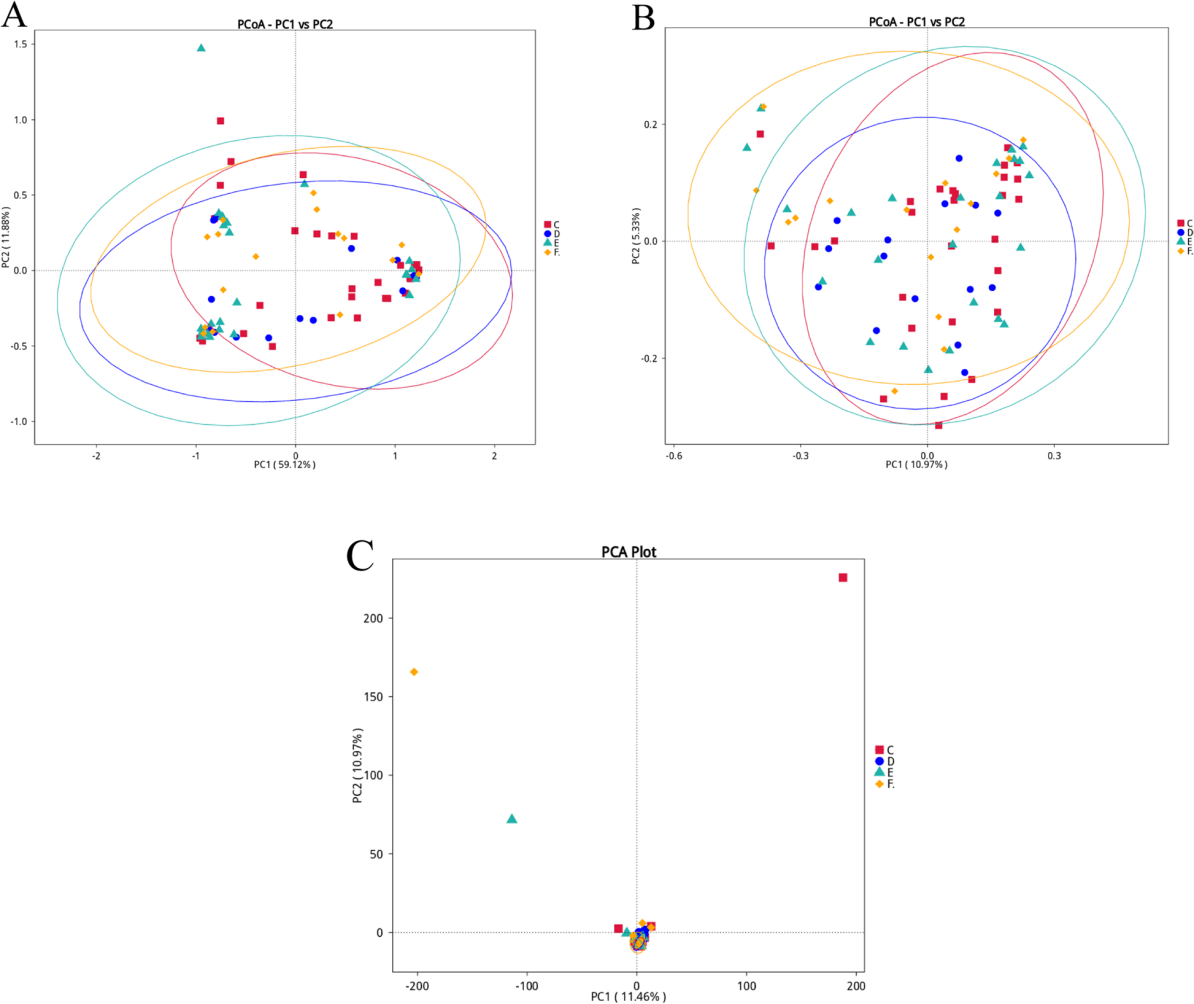
**A**Weighted Unifrac based distance from Principal Co-ordinates Analysis (PCoA) analysis. Horizontal coordinates indicate one principal component, vertical coordinates indicate another principal component, and percentages indicate the contribution of the principal component to the sample variance; each point in the graph indicates a sample, and samples from the same group are indicated using the same color (**B**) Unweighted Unifrac based distance from PCoA analysis. **C** Euclidean based distances from Principal Component Analysis (PCA) analysis. The horizontal coordinate indicates the first principal component, and the percentage indicates the contribution value of the first principal component to the sample difference; the vertical coordinate indicates the second principal component, and the percentage indicates the contribution value of the second principal component to the sample difference; each point in the graph indicates a sample, and samples in the same group are indicated using the same color; in PCA graphs with clustering circles, the clustering circle is added with the grouping information (clustering circles need more than 3 samples in the group)

The species distributions in the adenomyosis group and the control group were not completely separated, but were similar (Fig. 6).

**Fig. 7:**
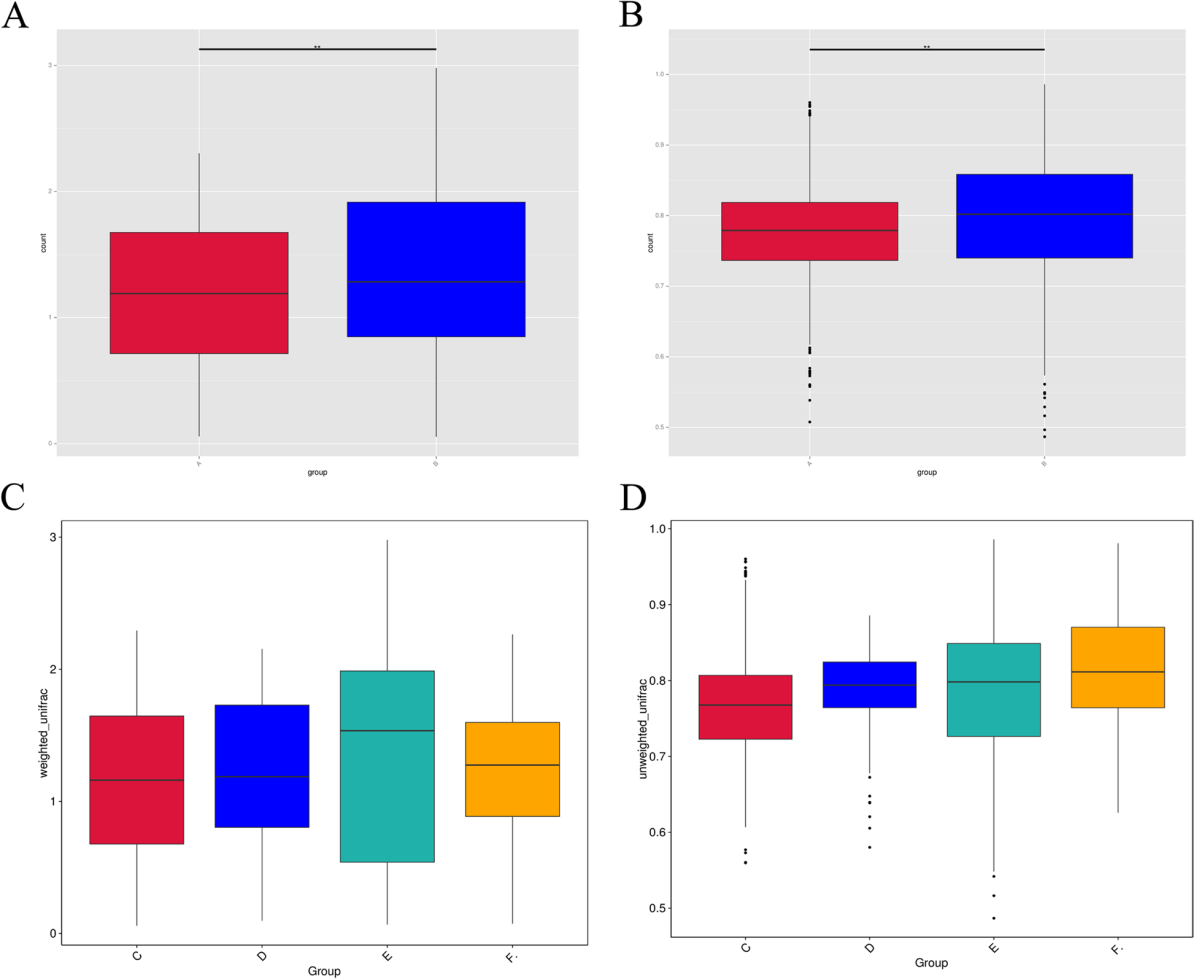
**A** Weighted Unifrac based distance from Beta-diversity analysis. **B** Unweighted Unifrac based distance from Beta-diversity analysis. The box plots of Beta-diversity between-group difference analysis can visualize the median, dispersion, maximum, minimum, and outliers of within-group sample similarity. At the same time, the T-test test was used to analyze whether the Beta diversity differences of species between groups were significant or not. **C** Weighted unifrac ased distance from Beta-diversity analysis during different menstrual cycles. **D** Unweighted unifrac ased distance from Beta-diversity analysis during different menstrual cycles

We analyzed the Beta-diversity index by using the t-test and found that the species Beta-diversity index was significantly different between the adenomyosis group and the control group (*p* = 0.018). However, based on Weighted Unifrac analysis, significant differences between the disease group and the control group were only observed throughout the luteal phase (*p* = *0.0146*) (Fig. 7 A, B, C, D).

*R* value was between (-1, 1), and *R* value was greater than 0, indicating that the difference between groups was greater than the difference within groups, which was significant (*P* < 0.05). The reasonableness of the grouping in this study was proved. (Table 2).

**Table 2:** Anosim analysis based on the Bray–Curtis distance. Anosim analysis is a non-parametric test used to test whether the difference between groups is significantly greater than the difference within groups, so as to determine whether the grouping is meaningful. We conducted the significance test of the difference between groups based on the rank of the Bray–Curtis distance value

Group	*R* value	*P* value
B-A	0.03067	0.044

**Fig. 8:**
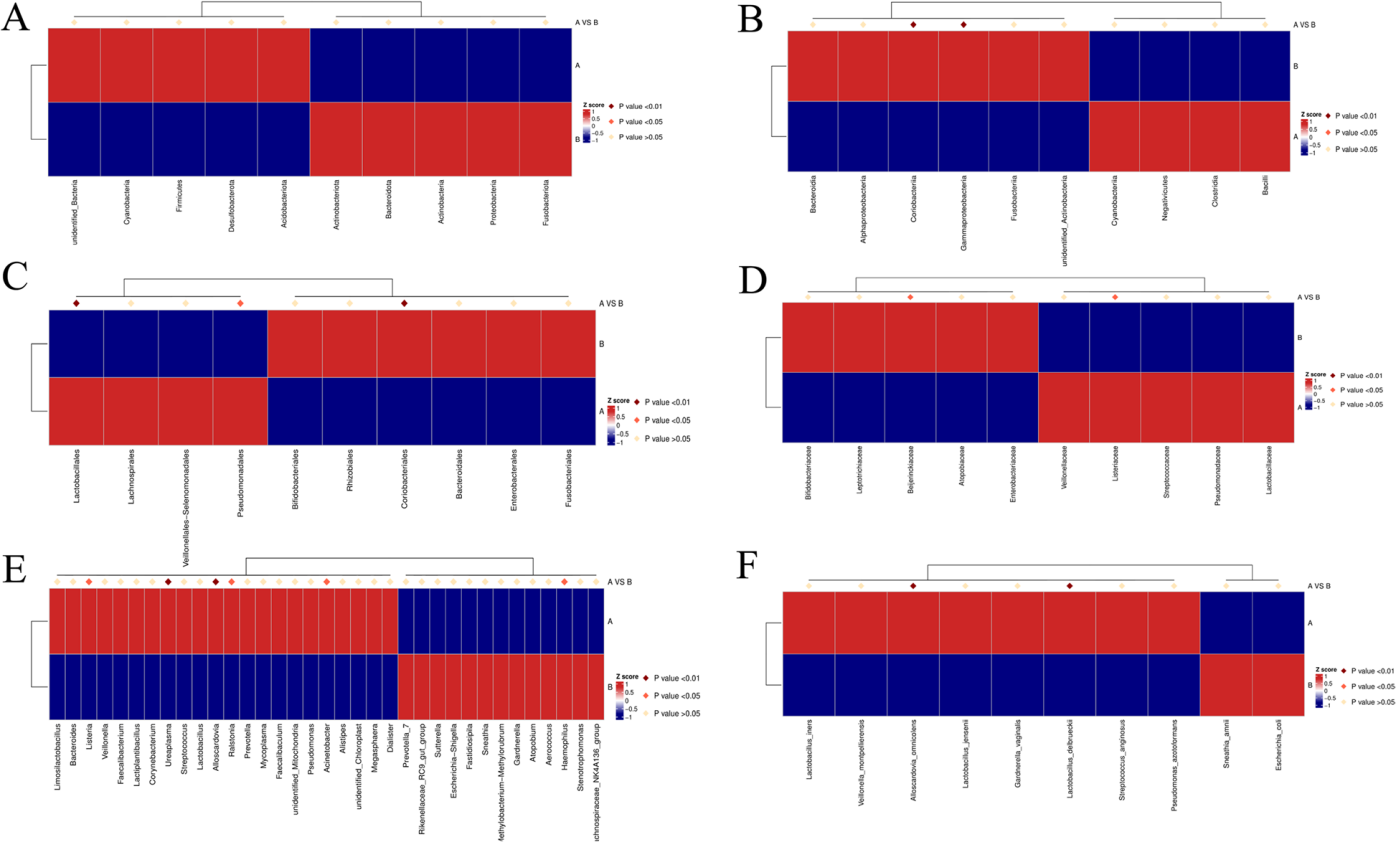
MetaStat analysis at (**A**) phylum, (**B**) class, (**C**) order, (**D**) family, (**D**) genus and (**E**) species level. For the species with significant differences between study groups, MetaStat method was used to screen the species with significant differences based on the species abundance tables of different levels

At the phylum level, there were no significant species differences between the adenomyosis group and the control group. At the class level the significant differences was in *Coriobacteriia* and *Gammaproteobacteria* (*p* < 0.01). At the class level the significant differences was in *Lactobacillales*, *Coriobacteriales* (*p* < 0.01),and in *Pseudomonadales* (*p* < 0.05). At the class level the significant differences was in *Beijerinckiaceae* and *Listeriaceae* (*p* < 0.05). At the genus level, that were in *Listeria*, *Ralstonia*, *Acinetobacter*, and *Haemophilus* (*p* < 0.01), and *Alloscardovia*,*Ureaolasma* (*p* < 0.05). Finally, at the species level,there was significant difference in *Alloscardovia_omnicolens* and *Lactobacillus_delbrueckii* (*p* < 0.01) (Fig. 8).

**Fig. 9:**
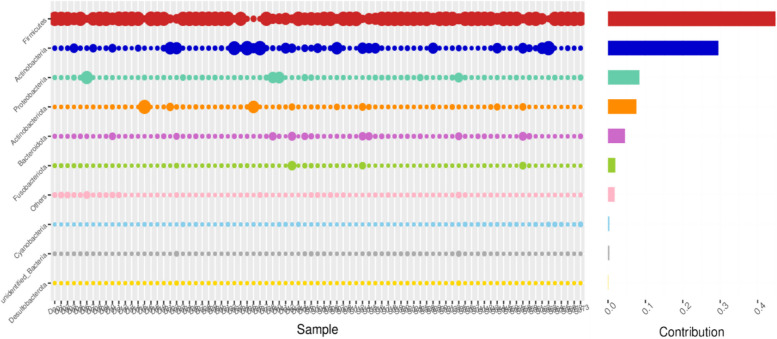
Simper analysis. It is a breakdown of the Bray–Curtis difference index that quantifies how much each species contributes to the difference between two groups. The results show the top 10 species with the highest contribution to the difference between the two groups and their abundance

At the phylum level, *Firmicutes* showed the highest species abundance in both the adenomyosis group and the control group, and at the same time, contributed the most to the species difference between the two groups (Fig. 9).

**Fig. 10:**
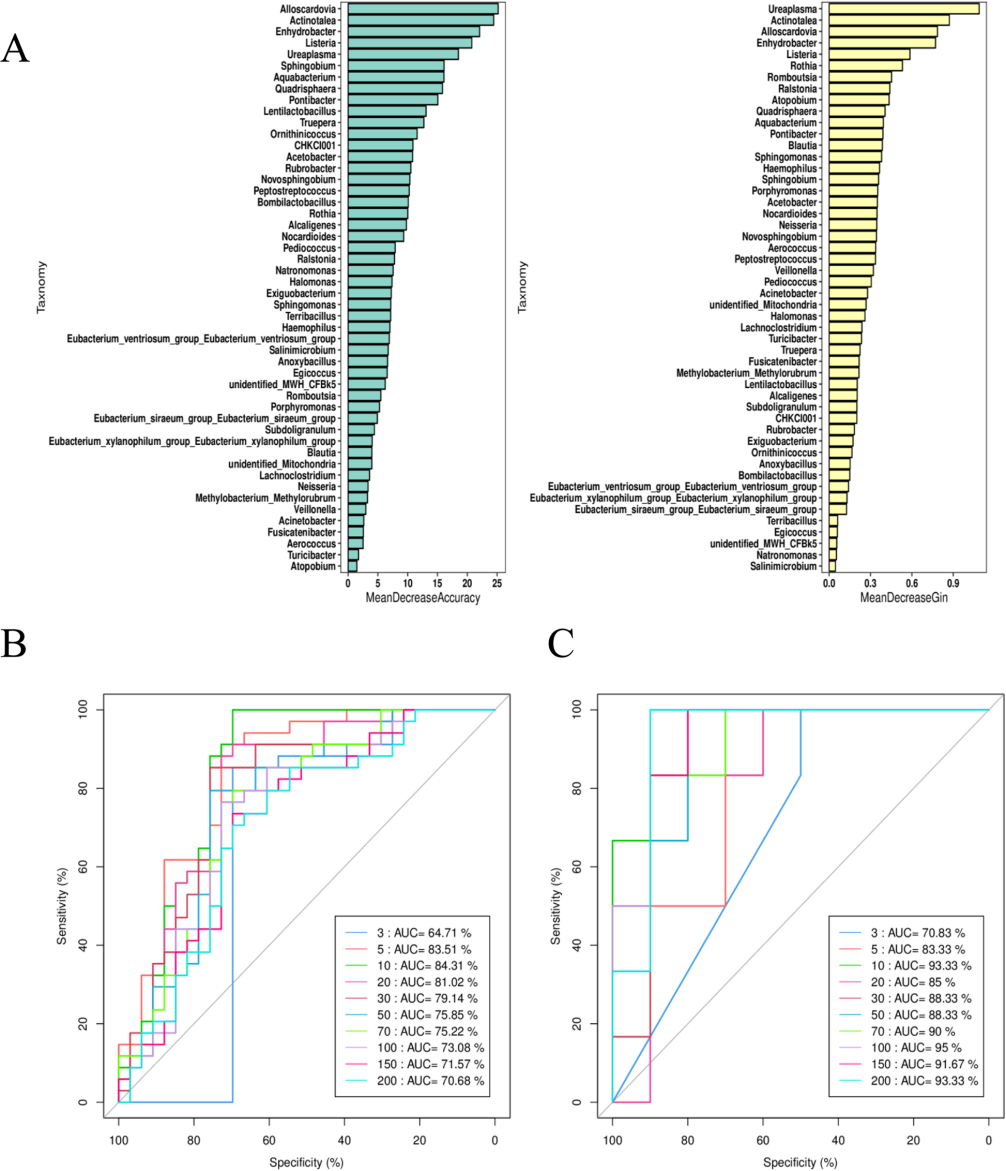
**A** MeanDecreaseAccuracy based analysis and MeanDecreaseGin based analysis. **B** proportion of false positive (Specificity), ordinate: proportion of true Sensitivity; (**C**) ROC curve of the test pair, abscess: proportion of false positive (Specificity), ordinate: proportion of true Sensitivity (specificity) Mean Decrease Accuracy measures the extent to which the prediction accuracy of random forest is reduced when the value of a variable is changed to a random number. The greater the value, the greater the importance of the variable. MeanDecreaseGini compared the importance of the variables by calculating the effect of each variable on the heterogeneity of the observed values at each node of the classification tree using the Gini index

Random forest is a classical machine learning model based on classification tree algorithm to screen features (biomarkers) that play an important role in classification or grouping. A default tenfold cross-validation was performed for each model, and Receiver Operating Characteristic Curve (ROC) curves were drawn to select potential Biomaker 50 as shown in Fig. 10.

## Discussion

Species diversity was analyzed using alpha diversity indices (Shannon index, chao1 index, ACE, and Simpson indices), and the number of microbial species and proportion of each species in a single sample were calculated. Results showed that species diversity of the two groups did not show significant differences, similar to the results of Chen et al. [19]. Although the species composition of the two groups was similar, species abundance significantly differed. At the phylum level, the relative abundance of Firmicutes was higher in the adenomyosis group than in the control group. At the genus level, except for the absolute species dominance of *Lactobacillus* in both groups, the relative abundance of *Gardnerella* in the adenomyosis group was significantly lower than that in the control group, which differed from the results of Kunaseth [20]. Other groups of vaginal bacilli were also detected, second only to *Lactobacillus* in overall abundance.

*Lactobacillus* vegetation in the female reproductive tract is critical for the maintenance of genital health. However, the exact pathogenesis of *Gardnerella vaginalis* remains unclear [21]. *Lactobacillus* and *Gardnerella* interact in the female reproductive tract; when the abundance of *Lactobacillus* decreases to a certain extent, the growth of *Gardnerella* can decrease or stop [22], and the imbalance of the two bacteria can change the acid–base environment of the vagina and produce mucosal adsorption and biofilm, promoting chronic, persistent infection and inflammation [23, 24]. A data analysis using the dominance network analysis framework found that *Lactobacillus* is not the dominant species in some healthy African women, and very few bacteria have a cooperative and mutually beneficial relationship with *Gardnerella* and *Lactobacillus iners *[25], contrary to previous views [26]. *L. iners* cooperate with *Gardnerella* but are inhibited by other species [27]. A high abundance of *Gardnerella* genomospecies indicate the presence of gene variants coding for virulence factors, such as cholesterol-dependent pore-forming cytotoxin vaginolysin and neuraminidase sialidase [28]. In this study, the abundance of *L. iners* in the adenomyosis group was found to be significantly higher than that in the control group, which was verified using the MetaStat method. Microbiomes from women diagnosed with Amsel-bacterial vaginosis (BV) were enriched for host immune response evasion and colonization functions by *L. iners*, and its role in the vaginal microbiome has been widely debated. A study has identified a specific set of *L. iners* genes associated with positive Amsel-BV diagnoses, and their data suggested that certain *L. Iners* strains may adhere to epithelial cells, contributing to the appearance of clue cells and becoming more difficult to displace in the vaginal environment [27]. In conclusion, the variation in *L. iners* and *Gardnerella* abundance may be a potential cause of adenomyosis, and maintaining the balance of Lactobacillus and *Gardnerella* in the body may be a self-mechanism to maintain the stability of vaginal microecology.

However, little is known about how the genital microbiota affects host immune function and regulates disease susceptibility. *Lactobacillus* imbalance and high ecological diversity may be closely related to the concentration of pro-inflammatory cytokines in genital organs [29]. Patients with adenomyosis show leukocyte infiltration in the endometrial functional layer, and the number of macrophages and natural killer (NK) cells increased [30, 31]. Transcriptional analysis showed that antigen-presenting cells sense gram-negative bacterial products in situ via Toll-like receptor 4 (TLR-4) signalling, promoting genital organ inflammation by activating the nuclear factor kappa-B (NF-κB) signalling pathway and recruiting lymphocytes through chemokine production [29]. Immune dysregulation is present in the ectopic endometrium of patients with adenomyopathy and manifests as elevated T Cell Immunoglobulin Domain and Mucin Domain-3/Galectin-9 (Tim-3/Gal-9) expression and differential RNA methylation [32, 33]. Therefore, we speculated that vaginal microecological changes affect the important role of Tim-3/Gal-9 in immunosuppression through some mechanism, causing the persistence of infection, affecting the growth environment of the endometrial tissue, and causing adenomyosis. In addition, the expression of Type I interferon (IFN-I) inducers is increased in the ectopic endometrium in adenomyosis. The increased levels of IFN-Is and expression of IFN-stimulating genes and pro-inflammatory cytokines in tissues may be related to host immunity under the influence of certain microorganisms [34]. Recent literature has suggested that microbiota-induced interferon activation does not require direct host-bacterial interaction but the remote transport of bacterial DNA into host cells via bacteria-derived membrane vesicles [35]. In contrast with our finding that the beta diversity index was significantly higher in the adenomyosis group than in the control group, the increased bacterial diversity in the vagina probably explains the activation of the host’s innate immune response in the ectopic endometrium in adenomyosis [5, 20]. Endometriosis and adenomyosis are closely related disorders. Their pathophysiology and clinical symptoms such as chronic pain are extremely similar [36]. There is a correlation in the microbial composition of both intestinal and cervicovaginal microbial niches, and over 50% overlap in species abundance and cell density [37]. Central sensitisation is known to be significantly involved in endometriosis-associated chronic pelvic pain [38]. Dysbiosis may potentially lead to incorrect immune responses, triggering the development of inflammatory pain [39], such as that seen in endometriosis and adenomyosis. All the patients with adenomyosis included in the study have obvious dysmenorrhea. However, further studies are may elucidate the association between microbial changes and chronic pain.

The microbiota of the female reproductive system is influenced by changes in age and system physiology, and the menstrual cycle is a major disruptor of the vaginal microbiome. Different microbiota characteristics are observed in women at different physiological stages [40]. In healthy women of reproductive age, the vaginal microbiome composition changes dramatically before and after menstruation [41]. Menstrual blood flowing through the vagina leaves sufficient iron necessary for pathogens, and the iron necessary for pathogen metabolism [42], which is reduced by the iron-binding affinity of lactoferrin, is replenished. Additionally, studies measuring oestradiol levels and vaginal microbiome composition in women who use oral contraceptives to inhibit ovulation have shown that the high diversity observed during menstruation is mainly driven by oestradiol withdrawal before menstruation rather than by the dynamic drive of progesterone. *Lactobacillus* abundance increases during the follicular and luteal phases, gradually normalising the vaginal microecology [41, 43]. Under the influence of this periodicity, combined with our test results, different types of dominant bacterial profiles were observed in patients with adenomyosis in both luteal and follicular stages, which provided a reference for the detection of biomarkers in patients with specific menstrual cycles or to evaluate their efficacy.

In summary, in this study, an increase in microbial richness was associated with adenomyosis, and the microbiome characteristics of patients with and without adenomyosis differed according to the menstrual cycle. This study has three notable limitations: 1) the final sample size was limited because of coronavirus disease 2019 (COVID-19), 2) large sample of clinical data for verification was not available, and 3) the different methods used in each study may have led to different conclusions. Furthermore, adenomyosis diagnosis remains unconfirmed without histological assessment. This may have led to misclassification in both cases (false positives) and controls (false negatives). In future research, we plan to develop standardized analysis software and large databases to continue our investigation of the mechanisms behind this association.

### Supplementary Information

Supplementary Material 1.

## Data Availability

The datasets generated and analyzed during the current study are available in the [China National Center of Bioinformation (CNCB)]database. The number of this project is CRA012802. [https://ngdc.cncb.ac.cn/gsub/submit/gsa/list].

## Abbreviations

CA125: Carbohydrate Antigen 125
PCR: Polymerase Chain Reaction
CTAB: Cetyltrimethylammonium Bromide.
OTUs: Operational Taxonomic Units
PCoA: Principal Co-ordinates Analysis
MRI: Magnetic resonance imaging
BV: Bacterial Vaginosis
NK cells: Natural killer
TLR-4: Toll-like receptor 4
NF-κB: Nuclear Factor kappa-B
Tim-3: T Cell Immunoglobulin Domain and Mucin Domain-3
GAL-9: Galectin-9
IFN-I: Type I interferon
COVID-19: Coronavirus disease 2019
