# Supplementary material for: Vaginal microbiome differences between patients with adenomyosis with different menstrual cycles and healthy controls
Source: BMC Microbiol. 2024 Jul 27;24:281. doi: 10.1186/s12866-024-03339-9 (PMC11282752; doi:10.1186/s12866-024-03339-9)
Supplement: Supplementary file 1 — Supplementary Material 1. [file 12866_2024_3339_MOESM1_ESM.docx]

| Sample name | Total reads | Combined  reads | Uncombined reads | Percent combined(%) | Combined_base(bp) | Min_len(bp) | Max_len  (bp) | Avg_len(bp) |
| --- | --- | --- | --- | --- | --- | --- | --- | --- |
| S001 | 195,193 | 192,773 | 2,420 | 98.76 | 81,934,089 | 45 | 430 | 425 |
| S004 | 208,658 | 208,654 | 4 | 100 | 88,186,721 | 56 | 430 | 423 |
| S005 | 211,474 | 210,068 | 1,406 | 99.34 | 89,902,285 | 34 | 430 | 428 |
| S006 | 207,435 | 205,966 | 1,469 | 99.29 | 87,938,296 | 37 | 430 | 427 |
| S007 | 201,636 | 201,614 | 22 | 99.99 | 84,026,933 | 100 | 430 | 417 |
| S008 | 231,545 | 230,688 | 857 | 99.63 | 98,646,931 | 37 | 430 | 428 |
| D001 | 215,514 | 214,388 | 1,126 | 99.48 | 91,493,335 | 12 | 430 | 427 |
| D002 | 234,808 | 232,133 | 2,675 | 98.86 | 98,424,469 | 20 | 430 | 424 |
| D003 | 202,255 | 201,807 | 448 | 99.78 | 85,489,673 | 51 | 430 | 424 |
| D004 | 149,870 | 149,325 | 545 | 99.64 | 63,013,566 | 12 | 430 | 422 |
| D006 | 178,747 | 176,926 | 1,821 | 98.98 | 75,487,948 | 51 | 430 | 427 |
| D007 | 167,233 | 164,791 | 2,442 | 98.54 | 69,183,771 | 12 | 430 | 420 |
| D008 | 212,098 | 210,966 | 1,132 | 99.47 | 89,814,551 | 53 | 430 | 426 |
| D009 | 200,954 | 200,822 | 132 | 99.93 | 85,754,085 | 12 | 430 | 427 |
| D010 | 176,526 | 174,056 | 2,470 | 98.6 | 74,390,826 | 12 | 430 | 427 |
| D011 | 168,008 | 167,709 | 299 | 99.82 | 70,884,814 | 50 | 430 | 423 |
| D012 | 206,106 | 204,577 | 1,529 | 99.26 | 87,395,460 | 71 | 430 | 427 |
| S011 | 82,823 | 82,360 | 463 | 99.44 | 35,249,033 | 101 | 430 | 428 |
| S012 | 89,118 | 87,063 | 2,055 | 97.69 | 37,334,447 | 52 | 430 | 429 |
| S014 | 87,245 | 73,989 | 13,256 | 84.81 | 30,730,221 | 52 | 430 | 415 |
| S015 | 81,493 | 80,142 | 1,351 | 98.34 | 33,746,259 | 12 | 430 | 421 |
| S016 | 71,703 | 59,550 | 12,153 | 83.05 | 24,961,564 | 12 | 430 | 419 |
| S017 | 90,461 | 76,937 | 13,524 | 85.05 | 32,842,718 | 12 | 430 | 427 |
| S018 | 66,651 | 52,233 | 14,418 | 78.37 | 22,262,506 | 12 | 430 | 426 |
| S019 | 86,155 | 82,253 | 3,902 | 95.47 | 35,183,455 | 50 | 430 | 428 |
| S020 | 86,448 | 84,832 | 1,616 | 98.13 | 36,221,309 | 52 | 430 | 427 |
| S021 | 90,690 | 83,135 | 7,555 | 91.67 | 35,429,006 | 12 | 430 | 426 |
| S024 | 88,924 | 80,810 | 8,114 | 90.88 | 34,518,993 | 51 | 430 | 427 |
| S025 | 87,177 | 85,065 | 2,112 | 97.58 | 36,346,930 | 10 | 430 | 427 |
| S045 | 65,192 | 50,798 | 14,394 | 77.92 | 21,740,914 | 12 | 430 | 428 |
| S055 | 82,787 | 82,410 | 377 | 99.54 | 35,271,958 | 46 | 430 | 428 |
| S059 | 58,736 | 56,428 | 2,308 | 96.07 | 24,022,048 | 12 | 430 | 426 |
| S061 | 86,723 | 84,355 | 2,368 | 97.27 | 36,047,778 | 12 | 430 | 427 |
| S062 | 67,964 | 52,307 | 15,657 | 76.96 | 21,964,272 | 12 | 430 | 420 |
| S063 | 83,992 | 83,085 | 907 | 98.92 | 34,025,525 | 12 | 430 | 410 |
| S068 | 64,464 | 55,441 | 9,023 | 86 | 23,719,422 | 49 | 430 | 428 |
| S072 | 92,710 | 90,161 | 2,549 | 97.25 | 38,588,826 | 12 | 430 | 428 |
| S073 | 87,963 | 80,446 | 7,517 | 91.45 | 34,307,615 | 12 | 430 | 426 |
| D014 | 91,764 | 70,381 | 21,383 | 76.7 | 30,151,388 | 12 | 430 | 428 |
| D015 | 67,693 | 54,751 | 12,942 | 80.88 | 23,328,302 | 12 | 430 | 426 |
| D018 | 94,084 | 92,272 | 1,812 | 98.07 | 39,538,613 | 12 | 430 | 429 |
| D019 | 90,146 | 88,620 | 1,526 | 98.31 | 37,874,748 | 12 | 430 | 427 |
| S030 | 78,731 | 63,989 | 14,742 | 81.28 | 27,416,389 | 46 | 430 | 428 |
| S031 | 77,001 | 57,443 | 19,558 | 74.6 | 24,617,459 | 12 | 430 | 429 |
| S042 | 96,064 | 94,136 | 1,928 | 97.99 | 40,206,358 | 10 | 430 | 427 |
| D021 | 67,217 | 50,370 | 16,847 | 74.94 | 21,509,975 | 12 | 430 | 427 |
| D022 | 80,148 | 77,739 | 2,409 | 96.99 | 32,225,895 | 51 | 430 | 415 |
| D023 | 57,794 | 50,899 | 6,895 | 88.07 | 21,054,633 | 12 | 430 | 414 |
| D026 | 80,534 | 78,608 | 1,926 | 97.61 | 33,628,725 | 12 | 430 | 428 |
| D028 | 92,191 | 91,893 | 298 | 99.68 | 39,356,562 | 45 | 430 | 428 |
| D029 | 83,786 | 65,283 | 18,503 | 77.92 | 27,944,626 | 12 | 430 | 428 |
| D030 | 60,135 | 54,601 | 5,534 | 90.8 | 23,395,252 | 53 | 430 | 428 |
| D031 | 79,677 | 62,681 | 16,996 | 78.67 | 26,809,456 | 13 | 430 | 428 |
| D033 | 89,488 | 88,254 | 1,234 | 98.62 | 36,485,877 | 53 | 430 | 413 |
| D034 | 92,365 | 91,922 | 443 | 99.52 | 39,235,902 | 52 | 430 | 427 |
| D035 | 81,378 | 79,905 | 1,473 | 98.19 | 32,892,738 | 53 | 430 | 412 |
| D037 | 66,559 | 55,342 | 11,217 | 83.15 | 22,760,627 | 12 | 430 | 411 |
| D038 | 62,419 | 53,441 | 8,978 | 85.62 | 22,825,298 | 12 | 430 | 427 |
| D040 | 55,911 | 51,678 | 4,233 | 92.43 | 22,095,047 | 52 | 430 | 428 |
| D043 | 68,717 | 58,427 | 10,290 | 85.03 | 24,254,238 | 10 | 430 | 415 |
| D046 | 71,299 | 65,101 | 6,198 | 91.31 | 27,580,858 | 12 | 430 | 424 |
| S058 | 76,801 | 76,521 | 280 | 99.64 | 31,834,029 | 62 | 430 | 416 |
| S028 | 77,085 | 75,764 | 1,321 | 98.29 | 32,426,294 | 12 | 430 | 428 |
| S033 | 85,549 | 82,343 | 3,206 | 96.25 | 35,091,395 | 12 | 430 | 426 |
| S035 | 82,179 | 81,005 | 1,174 | 98.57 | 33,857,692 | 43 | 430 | 418 |
| S037 | 90,011 | 89,271 | 740 | 99.18 | 38,284,026 | 52 | 430 | 429 |
| S064 | 79,298 | 65,441 | 13,857 | 82.53 | 27,972,216 | 12 | 430 | 427 |
| D016 | 93,584 | 80,197 | 13,387 | 85.7 | 32,954,023 | 12 | 430 | 411 |
| S029 | 86,229 | 85,014 | 1,215 | 98.59 | 35,792,747 | 12 | 430 | 421 |
| S036 | 77,871 | 65,281 | 12,590 | 83.83 | 27,845,280 | 12 | 430 | 427 |
| S041 | 94,766 | 91,785 | 2,981 | 96.85 | 39,201,535 | 12 | 430 | 427 |
| S043 | 88,150 | 85,323 | 2,827 | 96.79 | 36,414,164 | 12 | 430 | 427 |
| S044 | 82,098 | 81,157 | 941 | 98.85 | 34,088,777 | 12 | 430 | 420 |
| S050 | 87,452 | 86,284 | 1,168 | 98.66 | 36,955,252 | 12 | 430 | 428 |
| D013 | 88,557 | 88,431 | 126 | 99.86 | 37,839,226 | 12 | 430 | 428 |
| D024 | 85,615 | 84,141 | 1,474 | 98.28 | 35,912,494 | 12 | 430 | 427 |
| D027 | 96,064 | 94,742 | 1,322 | 98.62 | 40,416,480 | 45 | 430 | 427 |
| D032 | 86,410 | 84,711 | 1,699 | 98.03 | 36,278,486 | 51 | 430 | 428 |
| D036 | 89,650 | 88,450 | 1,200 | 98.66 | 36,352,757 | 51 | 430 | 411 |
| D041 | 93,908 | 92,180 | 1,728 | 98.16 | 38,214,593 | 12 | 430 | 415 |
| D042 | 84,949 | 84,310 | 639 | 99.25 | 35,509,362 | 50 | 430 | 421 |
| D044 | 86,109 | 84,687 | 1,422 | 98.35 | 36,277,228 | 50 | 430 | 428 |
| S065 | 83,763 | 82,140 | 1,623 | 98.06 | 35,199,686 | 51 | 430 | 429 |
| Total | 8,748,678 | 8,337,977 | 410,701 | 95.31 | 3,538,389,260 | 101 | 430 | 424 |

S1 Table Data for subsequent analysis
